# An in-depth analysis of research on posthepatectomy liver failure (2006-2024): exploring trends and future directions through a bibliometric approach

**DOI:** 10.3389/fmed.2025.1598579

**Published:** 2025-08-19

**Authors:** Yachen Wu, Jiangtao Li, Zhaohai Liu, Mengliang Jiang, Hao Liang, Sajid Ameer, Xilin Qu, Zhangtao Long, Zhu Zhu, Xiaoming Dai

**Affiliations:** The First Affiliated Hospital, Department of Hepatobiliary Surgery, Hengyang Medical School, University of South China, Hengyang, Hunan, China

**Keywords:** PHLF, hotspots, trends, bibliometric analysis, visualization analysis

## Abstract

**Objective:**

This study aims to identify global hotspots and future trends within the research on posthepatectomy liver failure (PHLF).

**Design:**

Bibliometric analysis through science mapping and performance analysis.

**Data sources:**

The Web of Science Core Collection.

**Data extraction and synthesis:**

The Web of Science Core Collection database was searched for literature related to PHLF from 2006 to 2024. The authors, publishing institutions, countries, cited literature, journals, and keywords of the included studies were utilized for bibliometric analysis using CiteSpace and VOSviewer.

**Results:**

The analysis included 986 publications authored by 296 researchers from 60 countries and 246 institutions across 292 journals. The most prolific authors were Aldrighetti Luca, Cescon Matteo and Sparrelid Ernesto. The institutions with the most publications were Naval Medical University, Ruprecht Karls University Heidelberg, and the University of Amsterdam. The countries with the largest number of publications were China, Japan, and the United States. The most commonly cited literature was “posthepatectomy liver failure: a definition and grading” by the International Study Group of Liver Surgery (ISGLS). Annals of Surgery ranked first in the number of co-citations among journals. Currently, the research hotspots of PHLF focus primarily on novel surgical procedures such as liver venous deprivation, associating liver partition and portal vein ligation for staged hepatectomy, and the prediction of PHLF.

**Conclusion:**

Our study elucidates the global research status of PHLF and clarifies the relevant research hotspots and trends, providing clinicians and researchers with a better understanding of the state of the art and directions for future research.

## 1 Introduction

Hepatic resection is an effective treatment for various benign and malignant liver diseases (1–3). During recent years, despite the increasing safety of hepatectomy (4–7), posthepatectomy liver failure (PHLF) has remained an unavoidable and serious postoperative complication. The prevalence of PHLF ranges from 8% to 12% (8), and it accounts for 2.5% of hepatectomy-related deaths (9). Severe PHLF, one of the major causes of early postoperative death following hepatectomy (10, 11), leads to prolonged patient hospitalization, increased healthcare costs, and decreased long-term survival (8, 12, 13). Therefore, in-depth studies on PHLF are crucial for its prevention and treatment.

Over the past decade, researchers have achieved advancements in the prediction and management of PHLF. Moreover, some reviews summarized the progress in the prevention, clinical management, and treatment of PHLF (14–18). Due to the differences in the timing of reporting and the themes focused on in previous reviews, the current status and hotspots of global research on PHLF remain unclear; thus, a systematic and comprehensive analysis is necessary. Bibliometrics, a method for studying existing literature and its citations, has been used to analyze and compare a large amount of literature quickly, objectively quantify and analyze data, thoroughly excavate the information underlying the literature, and reveal hidden patterns and associations. In addition, bibliometrics can both measure the academic impact of literature and reveal trends in the development of a discipline. However, no bibliometric studies of PHLF have been published to date.

In this study, we visualize and analyze the development history, spatial density, areas of research interest, and future directions of the research literature related to PHLF. Our study aims to examine the current state of research in this field and identify future research directions.

## 2 Materials and methods

### 2.1 Data retrieval

**FIGURE 1:**
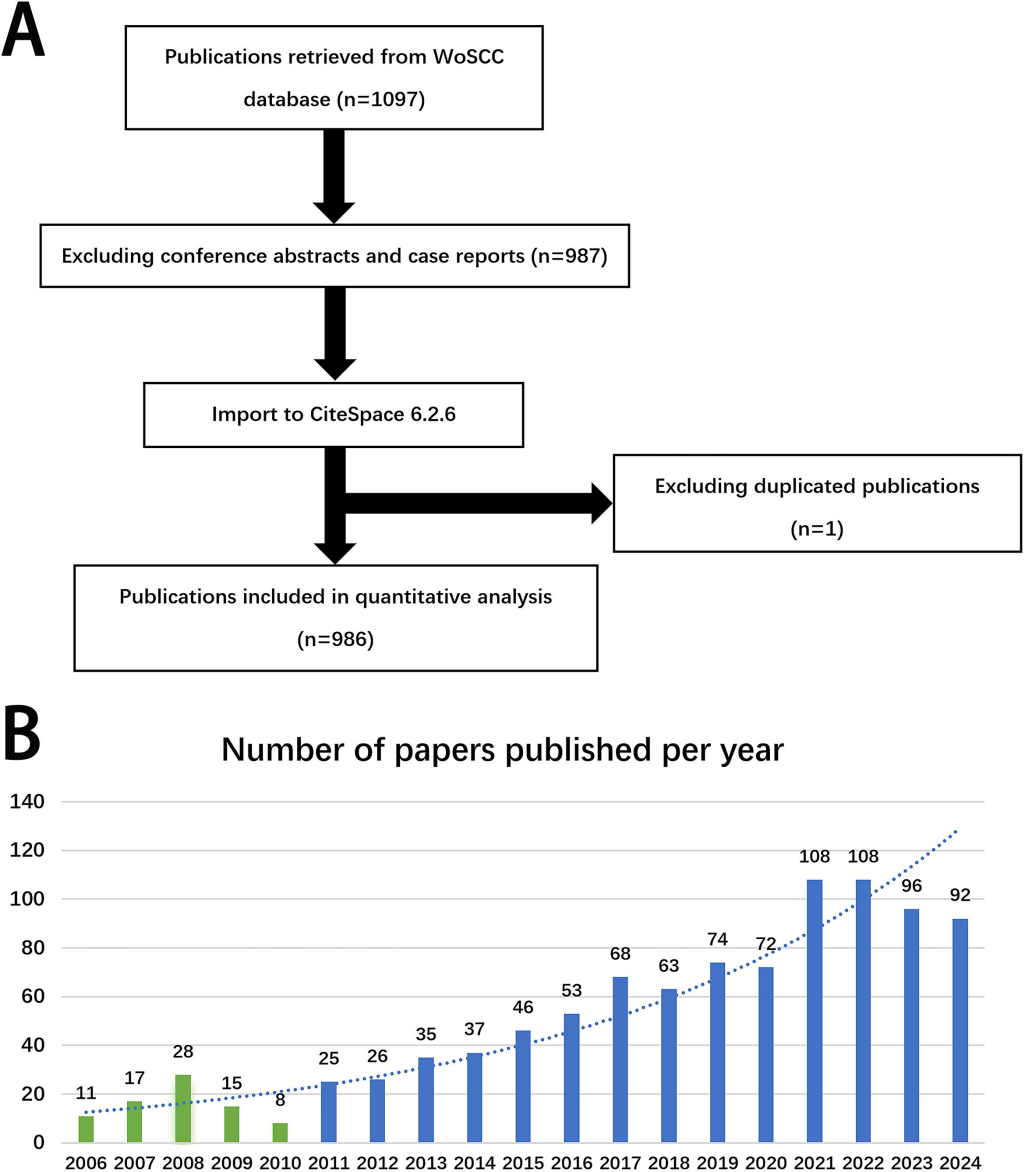
**(A)** Flowchart of literature selection. **(B)** Annual trends of articles on PHLF published from 2006 to 2024.

The Web of Science Core Collection was searched with the following search strategy: TS = (“posthepatectomy liver failure”) OR TS = (“post hepatectomy liver failure”) OR TS = (“postoperative liver failure”) OR TS = (“postoperative liver insufficiency”). A total of 1097 papers published up to December 31, 2024, were retrieved. After excluding conference abstracts and case reports, a total of 987 papers remained. These papers were exported in.txt format, deduplicated, and secondarily screened using CiteSpace. Ultimately, 986 articles were included in the current bibliometric analysis (Figure 1A).

### 2.2 Data analysis

The annual number of publications in the field of PHLF was summarized, and bar charts were drawn using Microsoft Excel. Data analysis and visualization were performed using CiteSpace 6.2.6 with parameters that utilized 1-year slices from 2006 to 2024. Visualization analysis was performed to generate a scientific knowledge atlas. Each node represents an author, institution, country, journal, reference, or keyword. A purple ring at a node indicates centrality, and centrality greater than or equal to 0.1 shows that the node has a significant impact on the field and should be considered a pivotal point. The more closely a node is connected to other nodes in the graph, the more centrally the country or institution represented by that node is. The line between nodes represents the cooperative relationship between countries or institutions, and the thickness of the line is proportional to the closeness of cooperation (19–21). The obtained full records and cited references were input into VOSviewer 1.6.20, and the corresponding parameters were set based on the analyzed contents to generate visual maps for collaborative network analysis.

## 3 Results

### 3.1 Annual number of publications related to PHLF

The annual number of publications related to PHLF roughly reflects the research hotspots and the development speed of the field. The number of related publications was low from 2006 to 2010. However, as knowledge about PHLF increased, the number of related publications began to grow annually since 2011 (Figure 1B).

### 3.2 Analysis of the authors of the published literature

**FIGURE 2:**
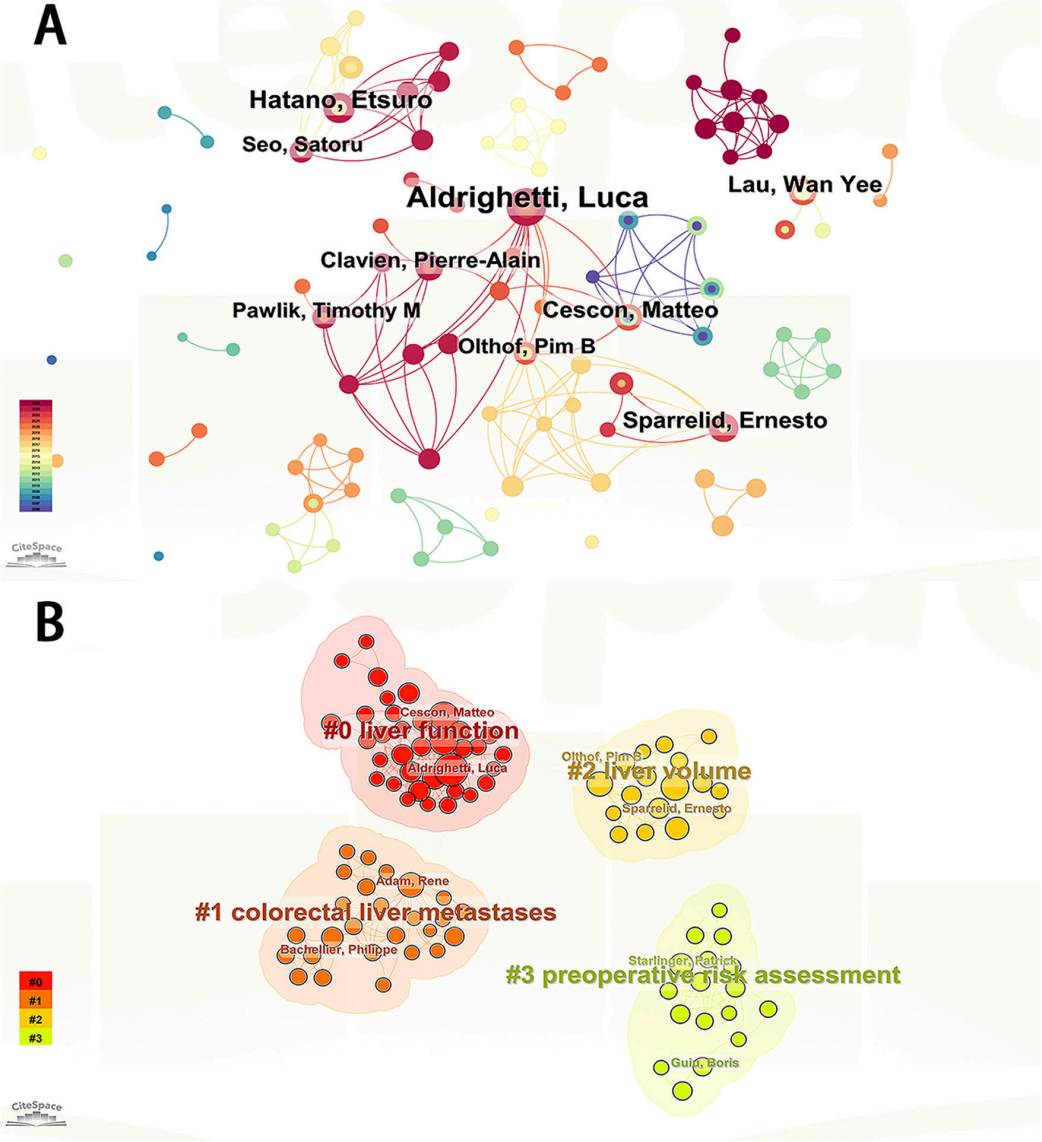
**(A)** Visualization of authors publishing research related to PHLF. **(B)** Visualization of authors clustering associated with PHLF.

Visual analysis was performed to examine authors by setting the minimum number of related studies to 10 (Figure 2A). The corresponding nodes represent individual authors; collaboration between authors is shown by lines connecting the nodes, with thicker lines indicating closer collaboration (22–24). The node representing Aldrighetti Luca was significantly larger than the other nodes, indicating that this author has the most publications. We found large node clusters centered on Aldrighetti Luca, Cescon Matteo, and Sparrelid Ernesto, as well as small node clusters concentrated on Hatano Etsuro and Taura Kojiro. The authors Aldrighetti Luca and Hatano Etsuro did not cluster together. The absence of line segments between the nodes showed that there was no communication between these authors, which might be related to their different locations and research subfields.

Author cluster analysis helps researchers gain a deeper understanding of the knowledge output in their research field, identify potential partners, and foster academic collaboration. Further cluster analysis of these authors revealed that their research directions were divided into four categories: liver function, liver volume, colorectal liver metastases, and preoperative risk assessment (Figure 2B). Table 1 lists the top ten authors in terms of the number of publications.

**TABLE 1:** The top 10 most prolific authors.

Rank	Authors	Centrality	Publications
1	Aldrighetti, Luca	0.07	20
2	Cescon, Matteo	0.06	14
3	Sparrelid, Ernesto	0.01	12
4	Hatano, Etsuro	0	12
5	Clavien, Pierre-Alain	0.06	12
6	Adam, Rene	0.08	10
7	Olthof, Pim B	0.11	10
8	Taura, Kojiro	0	10
9	Pawlik, Timothy M	0	9
10	Lau, Wan Yee	0	9

### 3.3 Analysis of institutions of published literature

**FIGURE 3:**
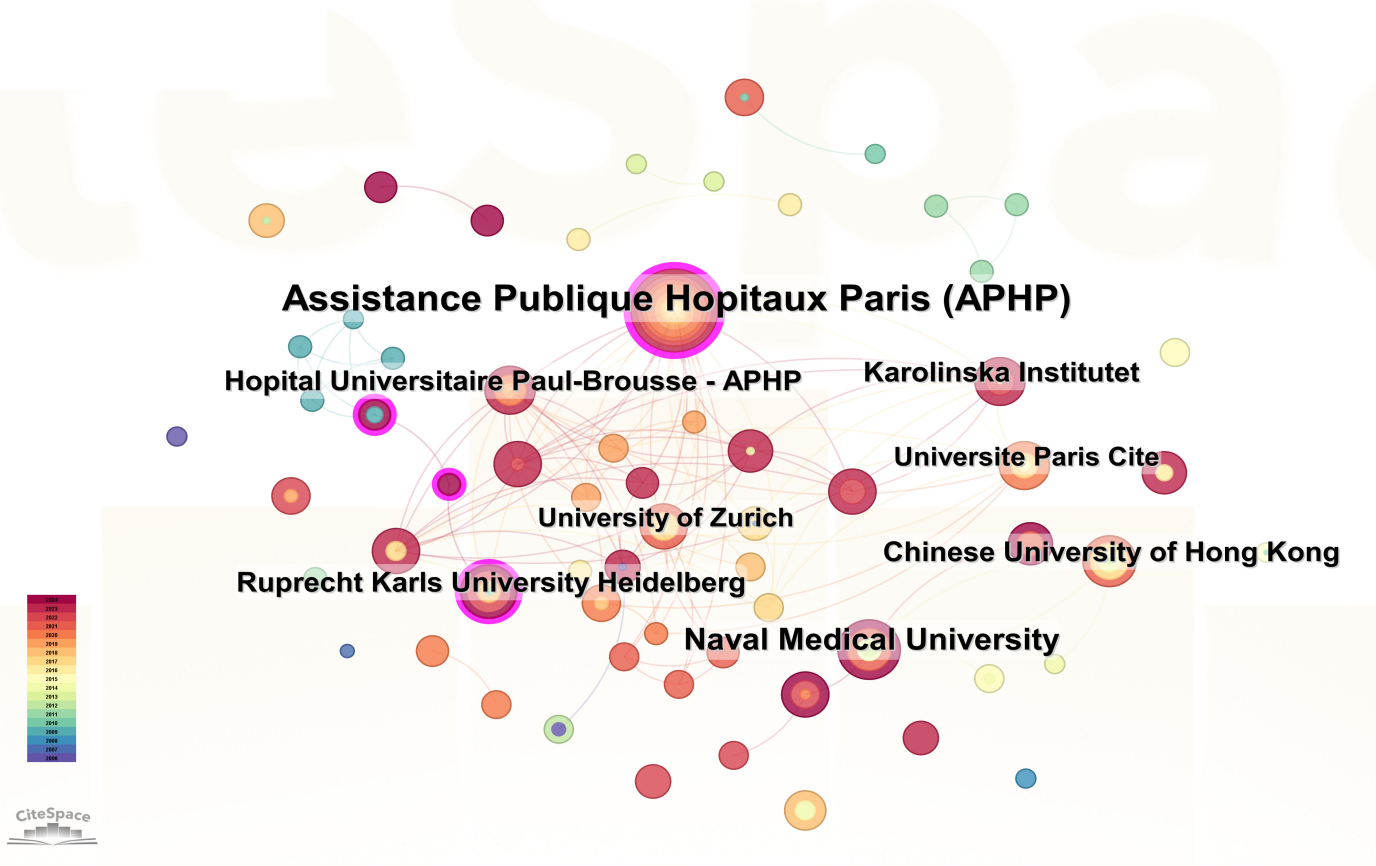
Visualization of institutions related to PHLF.

Visual analysis of institutions was performed by setting the minimum number of publications to 23 (Figure 3). A total of 246 institutions were represented in the publications related to PHLF. We selected the top 10 universities based on the number of publications for analysis (Table 2). Naval Medical University (*n* = 30), Ruprecht Karls University Heidelberg (*n* = 27), and the University of Amsterdam (*n* = 25) were the top three institutions in terms of the number of publications. Five of the top 10 institutions were located in Asia, including the Naval Medical University, the Chinese University of Hong Kong, Fudan University, Sichuan University, and Kyoto University. The remaining five institutions were in Europe, such as Ruprecht Karls University Heidelberg, University of Amsterdam, Vita-Salute San Raffaele University, University of Bologna, and University of Zurich. Centering on Assistance Publique Hopitaux Paris (APHP), organizations around the world with numerous publications have established good cooperative relationships (Figure 3). APHP and Hopital Universitaire Paul-Brousse are the top two institutions in centrality, with values of 0.2 and 0.14, respectively, indicating that these institutions are of high research importance in the field.

**TABLE 2:** The top 10 most prolific institutions.

Rank	Count	Centrality	Year	Institutions
1	30	0.05	2012	Naval Medical University
2	27	0.05	2011	Ruprecht Karls University Heidelberg
3	25	0.06	2010	University of Amsterdam
4	24	0.05	2012	Chinese University of Hong Kong
5	23	0.01	2014	Fudan University
6	23	0.04	2014	Vita-Salute San Raffaele University
7	22	0.03	2006	University of Bologna
8	22	0	2014	Sichuan University
9	20	0	2016	University of Zurich
10	19	0.01	2015	Kyoto University

### 3.4 Analysis of published literature by country

**FIGURE 4:**
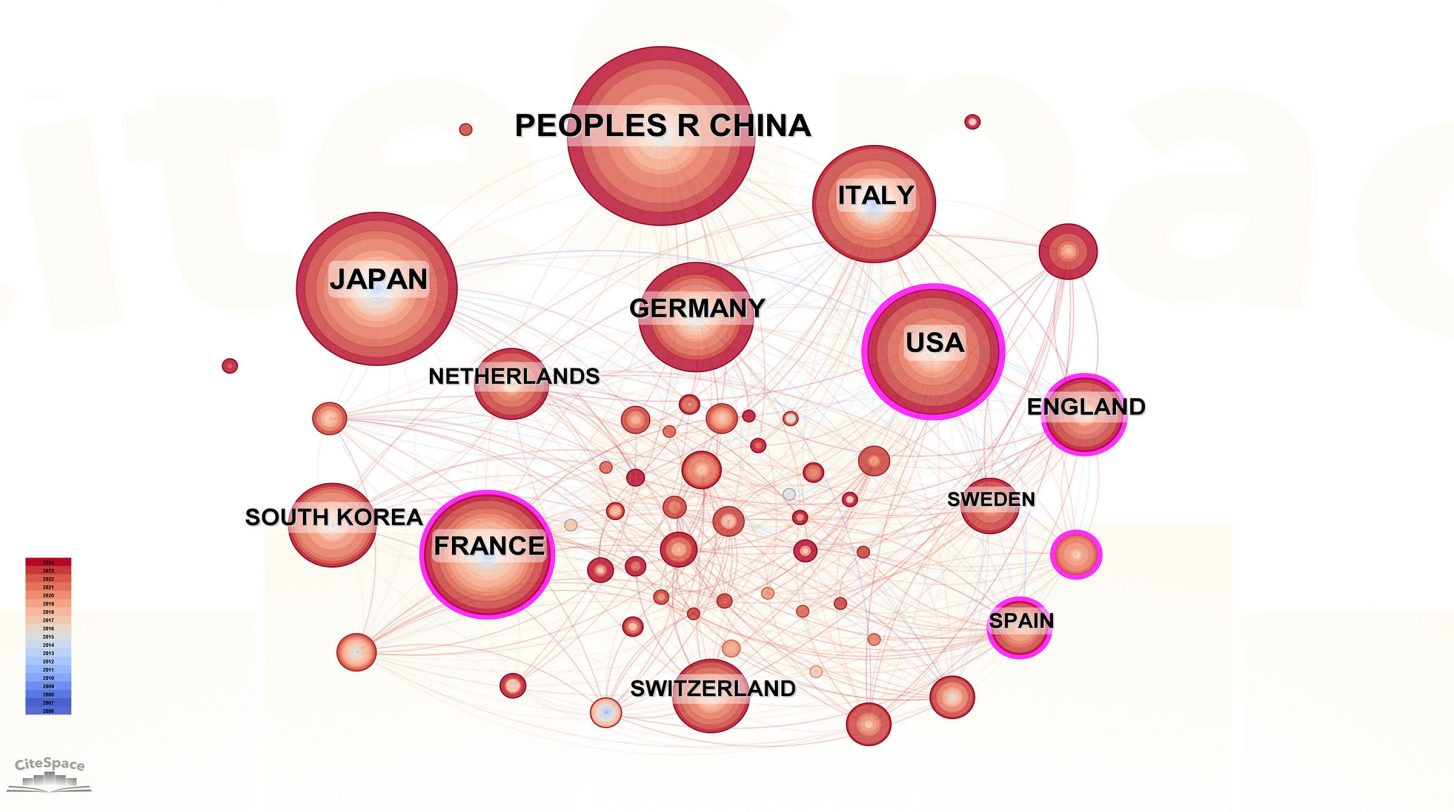
Visualization of countries related to PHLF.

A total of 60 countries were involved in the publication in this field, with China (*n* = 239), Japan (*n* = 188), and the United States (*n* = 131) ranking among the top three based on the number of publications. Therefore, these three countries have maintained a high level of research in this field. The top 10 countries with the most publications are in Asia (China, Japan, and South Korea, *n* = 481), Europe (Germany, Italy, France, the United Kingdom, Sweden, Netherlands, *n* = 447), and North America (the United States, *n* = 131) (Table 3). We used CiteSpace to construct a visual collaborative network map (Figure 4) and found that countries with a centrality value greater than 0.1 included the United States (0.23), France (0.18), Spain (0.16), Turkey (0.12), the UK (0.11), and Germany (0.1). Although Spain and Turkey are not among the top 10 most prolific countries, they rank high in centrality, suggesting that a country’s impact cannot be judged solely by the number of articles published. Each of the rings representing a country in the figure has lines connecting it to the other rings, suggesting that research in the field of PHLF is dominated by cooperation and communication between various countries. To further elucidate the contributions and academic influence of leading countries in the field of PHLF, we compiled a comparative table including the number of publications, local citations, global citations, and cooperation intensity. As shown in Supplementary Table 1, China and Japan lead in publication volume and citation impact, while international collaboration patterns vary significantly across regions.

**TABLE 3:** The top 10 most prolific countries.

Rank	Count	Centrality	Year	Countries
1	239	0.03	2006	Peoples R China
2	188	0.05	2006	Japan
3	131	0.23	2007	USA
4	106	0.1	2007	Germany
5	100	0.06	2006	Italy
6	95	0.18	2008	France
7	57	0.11	2007	England
8	54	0.02	2007	South Korea
9	46	0.02	2012	Switzerland
10	43	0.07	2007	Netherlands

### 3.5 Analysis of cited literature

**FIGURE 5:**
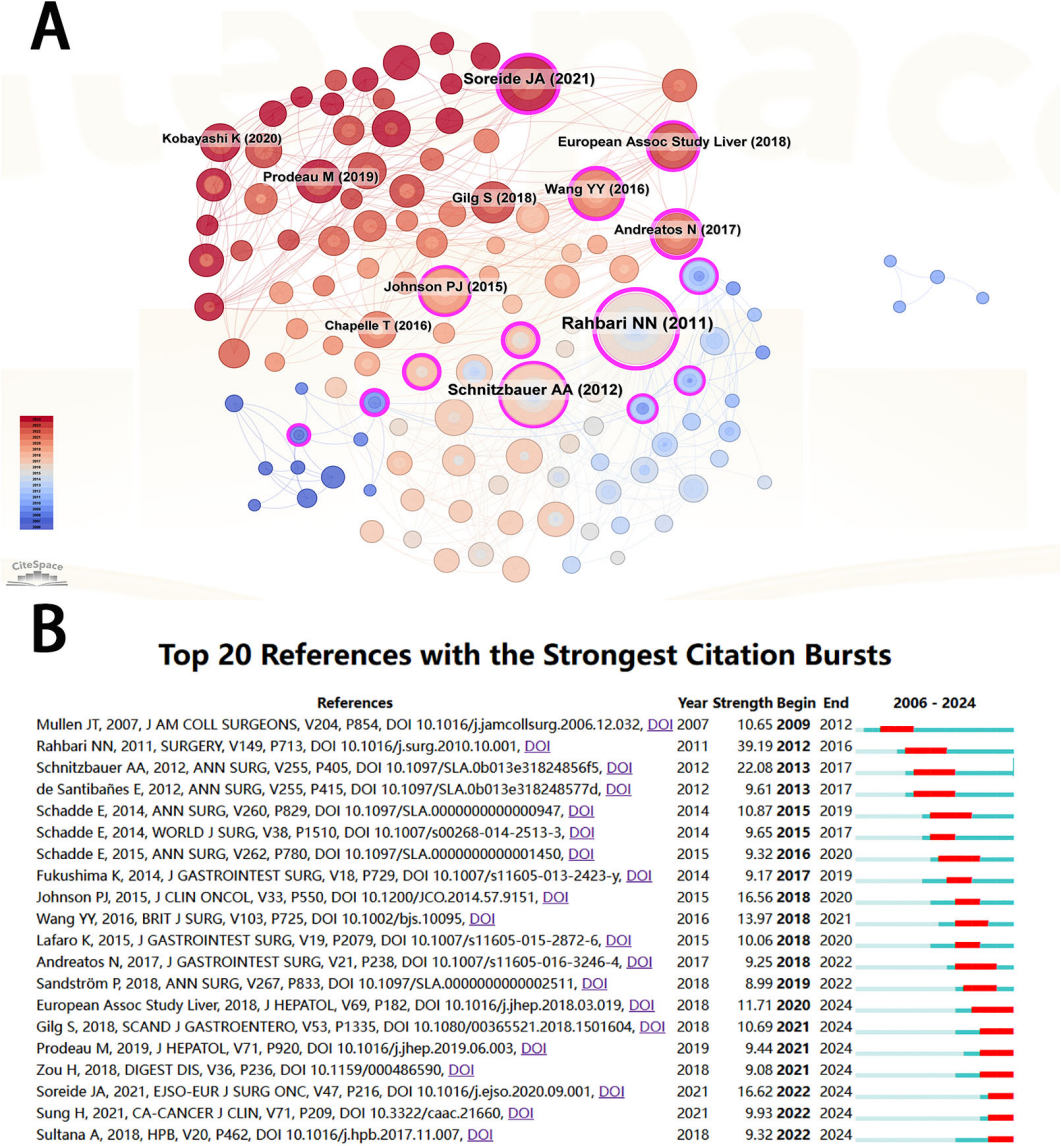
**(A)** Visualization of cited literature related to PHLF. **(B)** Cited literature with the strongest citation bursts related to PHLF from 2006 to 2024.

A total of 247 papers were cited in the included studies (Figure 5A), and the ten most cited papers are shown in Supplementary Table 2. The article entitled “posthepatectomy liver failure: a definition and grading by the International Study Group of Liver Surgery (ISGLS)” topped the list with a total of 80 citations. It was published in Surgery in 2011 (Journal Citation Reports Quarter 1, JCR Q1, impact factor = 3.8). Although the journal’s impact factor is not high, this article is a comprehensive review of 50 studies on PHLF by ISGLS, aiming to propose a unified definition and severity grading criteria for PHLF, as well as provide corresponding guidelines for clinical management. Three of the ten most cited articles were published in the Annals of Surgery. Figure 5B shows the most popular citations in this area over the years.

### 3.6 Analysis of cited and cocited journals

A total of 292 journals published relevant literature (Supplementary Table 3). The top three cited journals were Annals of Surgery (885 citations, JCR Q1, IF = 9), Surgery (795 citations, JCR Q1, IF = 3.8), and British Journal of Surgery (695 citations, JCR Q1, IF = 9.6).

**FIGURE 6:**
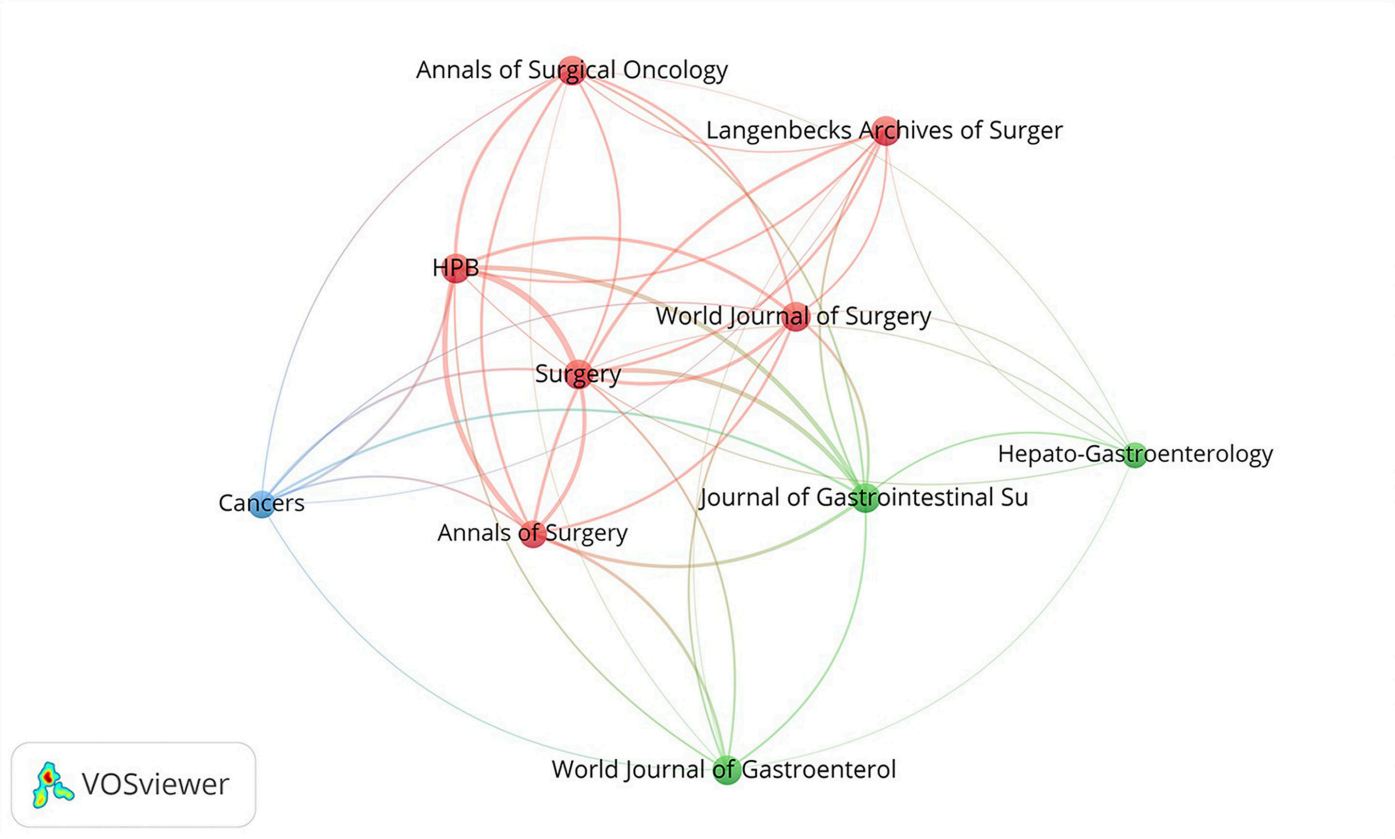
Visualization of top 10 cocited journal related to PHLF.

Cocited journal analysis provides a better understanding of the importance and impact of the relevant literature. As is shown in Figure 6, the top ten co-citation journals can be divided into three clusters, including red clusters (HPB, World Journal of Surgery, Surgery, Annals of Surgical Oncology, Langenbecks Archives of Surgery and Annals of Surgery), green cluster (Hepato-Gastroenterology, Journal of Gastrointestinal Surgery and World Journal of Gastroenterology) and blue cluster (Cancers).

### 3.7 Keyword analysis

**FIGURE 7:**
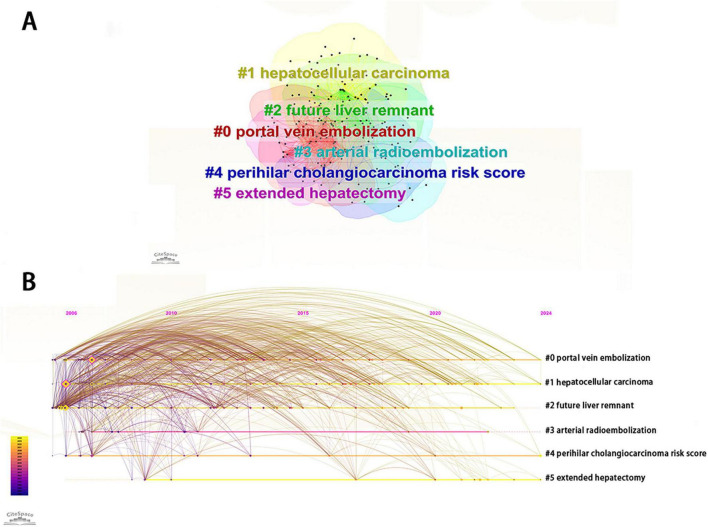
**(A)** Visualization of keyword clustering related to PHLF. **(B)** Visualization of keyword progression timeline related to PHLF from 2006 to 2024.

Through the cluster analysis of keywords, PHLF research can be subdivided into six clusters, including portal vein embolization, hepatocellular carcinoma, future liver remnant, arterial radioembolization, perihilar cholangiocarcinoma risk score, and extended hepatectomy (Figure 7A). Timeline view showed that portal vein embolization, hepatocellular carcinoma, perihilar cholangiocarcinoma risk score and extended hepatectomy were major research directions in the field of PHLF (Figure 7B).

**FIGURE 8:**
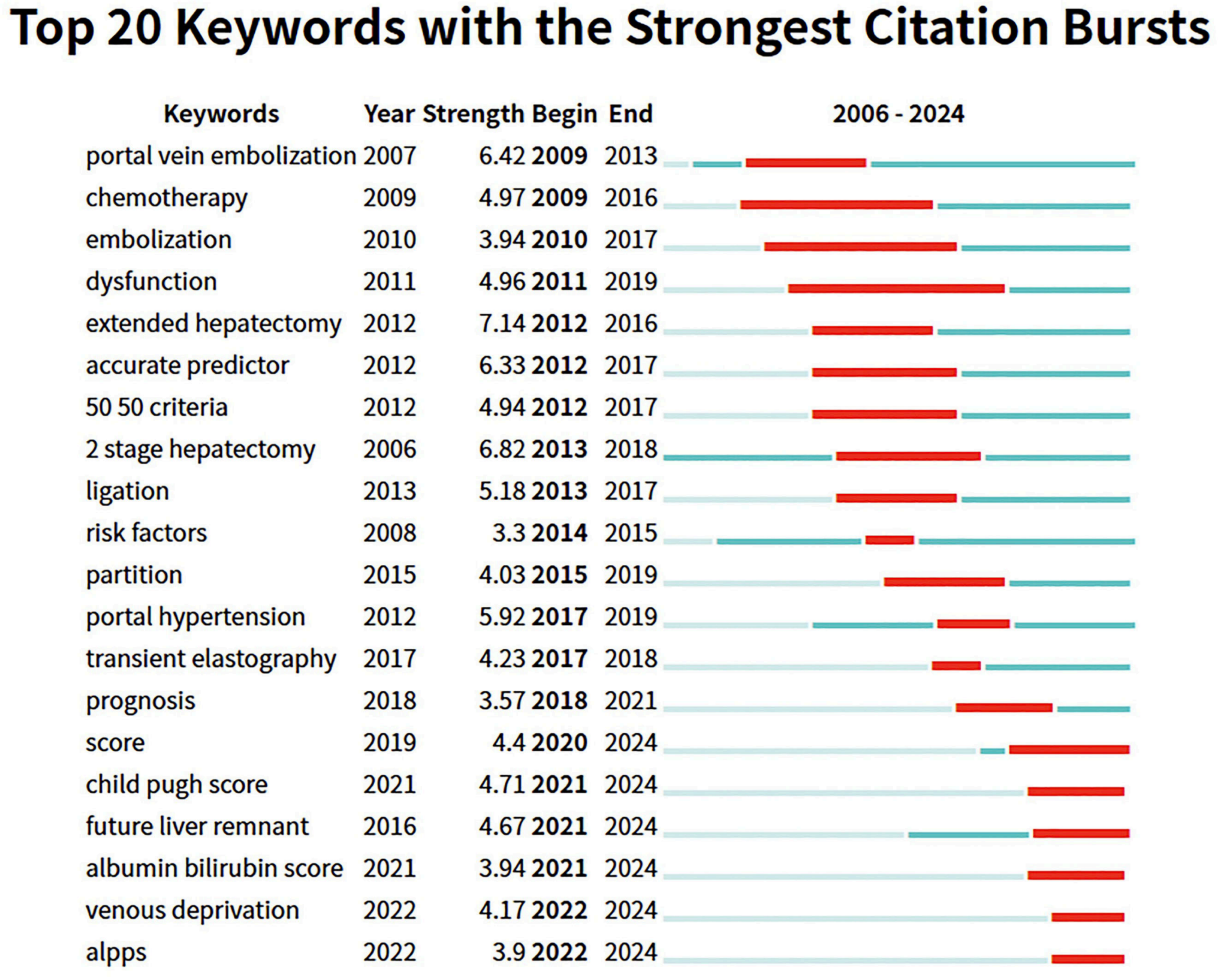
Keywords with the strongest citation bursts related to PHLF from 2006 to 2024.

To understand the changes in research hotspots, we further analyzed keywords from 2006 to 2024 using emergence analysis to identify the top 20 keyword citation bursts (Figure 8). We found that early studies primarily focused on translational therapy, including portal vein embolization, chemotherapy, and embolization. Then, the research direction shifted toward surgical treatment, including extended hepatectomy and two-stage hepatectomy. Nowadays, relevant studies focus on comprehensive assessments, such as the albumin-bilirubin score and the novel two-step hepatectomy, specifically liver venous deprivation (LVD) and associating liver partition and portal vein ligation for staged hepatectomy (ALPPS).

### 3.8 Ongoing clinical trials

Currently, six ongoing clinical trials on PHLF are classified into two categories: clinical intervention and risk prediction, with the latter dominating all the trials (Table 4).

**TABLE 4:** Ongoing clinical trials of PHLF.

NCT number	Categories	Method
NCT06300372	Intervention study	The effect of ultrasound-guided modified thoracoabdominal nerve plane block (M-TAPA) on PHLF
NCT05779098	Prediction model	The contents of VEGFA and PEDF in liver or serum were combined with the traditional pathophysiological index.
NCT04692259		Preoperative liver gadoxetate MRI
NCT06366048		Resected normal parenchymal volume (RNLV)
NCT06031818		VAE-MLP framework
NCT06181318		Quantitative MRI

## 4 Discussion

This study reviews the research progress in the field of PHLF from 2006 to 2024 and applies bibliometrics to visualize and analyze its development history, spatial density, areas of research interest, and future research directions. Bibliometric analysis offers a systematic approach to mapping research trends in PHLF, enabling the identification of emerging themes, highly cited publications, and key contributors within the field. Such insights are instrumental in elucidating the evolving focus on PHLF prediction, prevention, and management strategies, thereby informing future research directions and supporting the advancement of evidence-based clinical practices. To our knowledge, this is the first bibliometric study on the hotspots and trends of PHLF research. Our study presents a comprehensive knowledge map in the field of PHLF research, providing valuable references and insights for clinicians and researchers.

The article entitled “posthepatectomy liver failure: a definition and grading by the International Study Group of Liver Surgery (ISGLS),” which was published by Heidelberg University in Germany as a leading role, unified the definition and grading criteria for PHLF. The International Study Group on Liver Surgery (ISGLS), which published the latest definition and grading of PHLF, not only offered a theoretical basis for subsequent researchers but also promoted academic research and clinical practice in the field of PHLF worldwide (25). Moreover, some authors in ISGLS continued to explore and subsequently published relevant literature of great significance to the areas of prediction and assessment of PHLF. As a leading member of ISGLS, Heidelberg University provided a platform for related researchers to collaborate and publish several important guidelines to guide clinical research and practice in the field of PHLF, thereby providing authoritative references to researchers (26–28).

The three countries with the highest number of publications are China, Japan, and the United States. The number of related studies in these countries is closely related to the incidence of liver disease. Among them, patients in China and Japan have a high prevalence of hepatitis B and C virus-related liver diseases (e.g., cirrhosis and hepatocellular carcinoma) (29–32), whereas in the United States, non-alcoholic fatty liver disease is dominant (33, 34). Globally, China, Japan, and the United States account for 22% of the world’s population but 39.98% of the PHLF-related studies, indicating that these three countries have made considerable contributions to advancing research and development in the field of PHLF.

Visual analysis of keywords is beneficial for researchers to explore the current status and future trends, and their evolution reveals shifts in clinical practice and research focus. Regarding the technique of hepatectomy, early studies focused on “two-stage hepatectomy,” “extended hepatectomy,” and “portal vein embolization.” Recently, several novel surgical procedures have been emerging, including ALPPS and LVD. Notably, they play a crucial role in the prevention of PHLF by triggering the rapid hyperplasia of the future liver remnant (FLR), based on the mechanism by which they promote liver regeneration through the redistribution of liver blood flow and stimulation of a local inflammatory response (35–37). ALPPS is a two-stage surgical procedure that combines portal vein ligation with *in situ* liver partition, resulting in rapid FLR hypertrophy (up to 40–80%) within 7–10 days. This approach is feasible in carefully selected patients with preserved liver function and adequate performance status, but its technical complexity and high postoperative morbidity and mortality (reported up to 15% in early series) limit widespread adoption (38). In contrast, LVD is a minimally invasive, radiological technique that combines portal vein embolization (PVE) with simultaneous hepatic vein embolization to achieve significant FLR hypertrophy (40–60%) over 2–4 weeks. Compared to ALPPS, LVD is associated with lower morbidity, broader feasibility in patients with compromised liver function, and does not require surgery prior to resection. However, its longer hypertrophy interval raises concerns regarding potential tumor progression, and long-term oncologic outcomes remain under investigation (39). Both techniques offer substantial advantages over traditional PVE in selected cases, with ALPPS favoring rapid hypertrophy in aggressive tumors and LVD offering a safer, less invasive alternative for patients at higher surgical risk. Based on these findings, improvements in clinical practice should focus on refining patient selection and optimizing procedural strategies to enhance outcomes. The use of preoperative liver function assessment tools, such as indocyanine green retention rate, liver stiffness measurement, and hepatobiliary scintigraphy, may improve the identification of patients likely to benefit from ALPPS or LVD. Furthermore, technical modifications to ALPPS, including “partial ALPPS” or laparoscopic ALPPS, have demonstrated reduced complication rates and should be considered in high-risk patients. Standardization of LVD protocols and prospective randomized controlled trials is needed to establish its efficacy relative to PVE and its role in comparison to ALPPS. Ultimately, multidisciplinary collaboration among hepatobiliary surgeons, interventional radiologists, and oncologists is crucial for integrating these techniques effectively into oncologic treatment algorithms, ensuring optimal timing and sequencing of surgery and systemic therapy to enhance overall survival and disease-free outcomes. In the future, it is anticipated that a reduction in the incidence of PHLF and an improvement in the survival benefits for patients will be achieved through technical advancements and individualized treatment strategies.

Due to the lack of universally recognized methods, accurately predicting PHLF remains a challenge, which has emerged as a research hotspot. Clinical risk scores based on blood tests, including scoring systems such as Model for End-Stage Liver Disease (MELD), Fibrosis Index Based on the 4 factors (FIB-4), Albumin-Bilirubin Score (ALBI), and Aspartate aminotransferase to Platelet Ratio Index (APRI), are widely used to assess liver function reserve (40). These scoring systems are advantageous due to their reliance on readily available laboratory parameters, ease of calculation, and cost-effectiveness, which supports their routine implementation in clinical practice. However, they have several limitations in predicting PHLF with poor predictive ability (41–43). Recently, artificial intelligence (AI) has been utilized in a series of studies in this field (44, 45). Compared with clinical risk scores, AI is capable of integrating multiple types of data through various algorithms to generate a comprehensive model with better prediction performance. In terms of ongoing clinical trials, the majority are related to the prediction of PHLF using AI. Although preliminary results are encouraging, several limitations continue to restrict the clinical applicability of AI-based models. Most current models are trained on retrospective, single-center datasets with limited sample sizes, raising concerns about generalizability. The lack of standardized input features—ranging from laboratory markers to radiological parameters—further hampers reproducibility and model comparison across studies. A major barrier to clinical adoption lies in the limited explainability of many AI algorithms, which undermines clinicians’ confidence in model predictions and impedes integration into decision-making workflows. Moreover, few models have undergone robust external validation or prospective testing in real-world settings. Addressing these challenges will require collaborative efforts to develop multicenter, standardized datasets, incorporate explainable AI frameworks, and ensure rigorous clinical validation to support the safe and effective translation of these innovations into surgical practice.

Although progress has been made in understanding PHLF, current research remains insufficiently contextualized within the broader field of hepatobiliary diseases, such as hepatocellular carcinoma and cholangiocarcinoma (46, 47). These malignancies have benefited from decades of systematic investigation, supported by large-scale multicenter collaborations and the development of robust frameworks encompassing pathophysiology, diagnosis, prognosis, and treatment. In contrast, PHLF remains underexplored across multiple dimensions, characterized by a lack of standardized models, limited prospective validation, and fragmented efforts in both clinical and translational research. This disparity underscores a pressing need for targeted investment and methodological refinement, informed by the advances achieved in hepatobiliary oncology. Several critical gaps persist in the current PHLF literature. Long-term outcomes and survivorship in patients with PHLF are poorly characterized, limiting our understanding of its sustained clinical burden. Molecular and genetic predictors remain largely unexplored, despite their potential to refine risk assessment and support individualized treatment strategies. Finally, the integration of advanced analytical approaches—particularly artificial intelligence and machine learning—into PHLF prediction and decision support remains at an early stage (48–50). Future research must adopt multidisciplinary, data-driven strategies to address these limitations and enhance both prognostication and patient outcomes in hepatic surgery.

This study has several shortcomings. First, the analysis relied exclusively on the Web of Science Core Collection as the data source, while publications indexed in other major databases, such as Scopus, PubMed, and Embase, were not included. In particular, clinically relevant studies, guideline documents, or region-specific research not indexed in WoS might have been underrepresented, potentially limiting the comprehensiveness of the analysis. Second, the literature analyzed was restricted to English-language publications, which may limit the global representation of research on this topic and risk overlooking region-specific perspectives or culturally relevant findings. To address these issues, future studies should expand the scope of data sources by incorporating multiple bibliographic databases and including non-English language literature to achieve a more comprehensive and balanced analysis.

## 5 Conclusion

This study summarizes the characteristics of research in the field of PHLF, including authors, institutions, countries, references, and journals. We found an overall upward trend in the number of relevant publications from 2006 to 2024, with Europe and Asia being the main sources for major publishing organizations. For PHLF research, close collaboration between countries, institutions and authors has developed. Currently, studies related to PHLF are focusing on novel surgical procedures to overcome PHLF and prediction for the risk of PHLF. In conclusion, this study provides an overview of research in the field of PHLF and identifies new perspectives for the future.

## Strengths and limitations of this study

Bibliometric analysis was used to explore the global research landscape of posthepatectomy liver failure (PHLF).

This study investigates various elements of publications in the field of PHLF, including prolific authors, publishing institutions, countries, frequently cited literature, journals, and important keywords, which offer clinicians and researchers a better understanding of research hotspots and trends.

Using a single database or language might result in a selection bias that underestimates the output in this research field.

## Data Availability

The original contributions presented in this study are included in this article/Supplementary material, further inquiries can be directed to the corresponding authors.
