# Supplementary material for: An in-depth analysis of research on posthepatectomy liver failure (2006-2024): exploring trends and future directions through a bibliometric approach
Source: Front Med (Lausanne). 2025 Aug 19;12:1598579. doi: 10.3389/fmed.2025.1598579 (PMC12401975; doi:10.3389/fmed.2025.1598579)
Supplement: Supplementary file 1 [file Table_1.docx]

***Supplementary Material***

**Supplementary Table S1. Comparison of research metrics among the top 10 contributing countries**

| Rank | Countries | Publications | Local Citations | Global Citations | Cooperation  Intensity* |
| --- | --- | --- | --- | --- | --- |
| 1 | PEOPLES R CHINA | 239 | 399 | 5469 | Low |
| 2 | JAPAN | 188 | 200 | 4699 | High |
| 3 | USA | 131 | 98 | 4874 | Low |
| 4 | GERMANY | 106 | 179 | 3508 | High |
| 5 | ITALY | 100 | 147 | 4799 | High |
| 6 | FRANCE | 95 | 255 | 4318 | High |
| 7 | ENGLAND | 57 | 51 | 3446 | High |
| 8 | SOUTH KOREA | 54 | 51 | 1324 | Low |
| 9 | SWITZERLAND | 46 | 138 | 1428 | High |
| 10 | NETHERLANDS | 43 | 44 | 1683 | High |

*Cooperation intensity reflects the frequency of co-authorship and international collaboration.
