# Supplementary material for: An in-depth analysis of research on posthepatectomy liver failure (2006-2024): exploring trends and future directions through a bibliometric approach
Source: Front Med (Lausanne). 2025 Aug 19;12:1598579. doi: 10.3389/fmed.2025.1598579 (PMC12401975; doi:10.3389/fmed.2025.1598579)
Supplement: Supplementary file 2 [file Table_2.docx]

***Supplementary Material***

**Supplementary Table S2. Top 10 most cited references**

| Rank | Count | Centrality | Year | Cited Reference |
| --- | --- | --- | --- | --- |
| 1 | 79 | 0.44 | 2011 | DOI 10.1016/j.surg.2010.10.001 |
| 2 | 54 | 0.23 | 2012 | DOI 10.1097/SLA.0b013e31824856f5 |
| 3 | 41 | 0.14 | 2021 | DOI 10.1016/j.ejso.2020.09.001 |
| 4 | 36 | 0.02 | 2018 | DOI 10.1016/j.jhep.2018.03.019 |
| 5 | 32 | 0.09 | 2016 | DOI 10.1002/bjs.10095 |
| 6 | 31 | 0.02 | 2015 | DOI 10.1200/JCO.2014.57.9151 |
| 7 | 30 | 0.07 | 2019 | DOI 10.1016/j.jhep.2019.06.003 |
| 8 | 29 | 0.25 | 2017 | DOI 10.1007/s11605-016-3246-4 |
| 9 | 29 | 0.23 | 2014 | DOI 10.1097/SLA.0000000000000947 |
| 10 | 27 | 0.03 | 2018 | DOI 10.1080/00365521.2018.1501604 |
