# Supplementary material for: An in-depth analysis of research on posthepatectomy liver failure (2006-2024): exploring trends and future directions through a bibliometric approach
Source: Front Med (Lausanne). 2025 Aug 19;12:1598579. doi: 10.3389/fmed.2025.1598579 (PMC12401975; doi:10.3389/fmed.2025.1598579)
Supplement: Supplementary file 3 [file Table_3.docx]

***Supplementary Material***

**Supplementary Table S3.** **Top 10 most cited journals and Co-cited journals**

| Rank | Journal | Counts | | JCR  (2024) | | IF  (2024) | | Co-cited journal | Counts | JCR  (2024) | IF  (2024) |
| --- | --- | --- | --- | --- | --- | --- | --- | --- | --- | --- | --- |
| 1 | ANN SURG | | 885 | | Q1 | | 9.0 | ANN SURG | 4244 | Q1 | 9.0 |
| 2 | SURGERY | | 795 | | Q1 | | 3.8 | SURGERY | 1764 | Q1 | 3.8 |
| 3 | BRIT J SURG | | 659 | | Q1 | | 9.6 | HEPATOLOGY | 1525 | Q1 | 13.5 |
| 4 | HEPATOLOGY | | 594 | | Q1 | | 13.5 | BRIT J SURG | 1430 | Q1 | 9.6 |
| 5 | J AM COLL SURGEONS | | 573 | | Q1 | | 5.2 | HPB | 1251 | Q1 | 2.9 |
| 6 | HPB | | 566 | | Q2 | | 3.2 | J AM COLL SURGEONS | 1056 | Q1 | 5.2 |
| 7 | J GASTROINTEST SURG | | 551 | | Q1 | | 2.6 | J GASTROINTEST SURG | 1002 | Q2 | 3.2 |
| 8 | WORLD J SURG | | 524 | | Q1 | | 2.9 | WORLD J SURG | 951 | Q1 | 2.6 |
| 9 | J HEPATOL | | 422 | | Q1 | | 25.7 | J HEPATOL | 850 | Q1 | 25.7 |
| 10 | ANN SURG ONCOL | | 420 | | Q1 | | 3.7 | ANN SURG ONCOL | 702 | Q1 | 3.7 |
